# The Utility of the Oncotype DX Test for Breast Cancer Patients in an Australian Multidisciplinary Setting

**DOI:** 10.1155/2022/1199245

**Published:** 2022-01-31

**Authors:** Joseph Do Woong Choi, T. Michael D. Hughes, Gavin Marx, John Boyages, Josie Rutovitz, Csilla Hasovits, Andrew Parasyn, Senarath Edirimanne, Nicholas K. Ngui

**Affiliations:** ^1^Division of Surgery, Sydney Adventist Hospital, Sydney, New South Wales, Australia; ^2^Breast Multidisciplinary Team, Sydney Adventist Hospital, Sydney, New South Wales, Australia; ^3^Sydney Adventist Hospital Clinical School, Australian National University, Canberra, Australian Capital Territory, Australia; ^4^Department of Medical Oncology, Sydney Adventist Hospital, Sydney, New South Wales, Australia; ^5^ICON Cancer Center, Sydney Adventist Hospital, Sydney, New South Wales, Australia; ^6^The University of Sydney, Sydney, New South Wales, Australia

## Abstract

**Introduction:**

The Oncotype DX test is a genomic assay that generates a Recurrence Score (RS) predicting the 10-year risk of recurrence and response to adjuvant chemotherapy in ER+/HER2− breast cancer patients. The aims were to determine breast cancer distant recurrence and correlate with adjuvant chemoendocrine prescribing patterns based on the Oncotype DX recurrence score.

**Methods:**

We conducted a retrospective single-institution case series of 71 patients who had Oncotype DX assay testing after definitive surgery between 2012 and 2016. Both node-positive and node-negative patients were included. Patients were divided into Oncotype DX low risk (RS < 11) (n = 10, 14%), intermediate risk (RS 11–25) (n = 45, 63%), and high risk (RS > 25) (n = 16, 23%). Median follow-up was 6.1 years (range 4–8.9 years). Adjuvant treatment regimens and oncological outcomes were determined*. Results*. Mean age at diagnosis was 56 years (range, 33–77). Invasive ductal carcinoma (IDC) accounted for the majority (87%), with most tumors measuring between 10–20 mm (52%). 48% of the cohort were node positive. 15 of 16 high-risk patients (94%) received chemotherapy. 96% of intermediate-risk patients received endocrine therapy alone, one patient received chemoendocrine therapy (2%), and one declined systemic therapy (2%). In the low-risk group, 100% received endocrine therapy only. The high-risk group had the lowest mean ER% (*P* < 0.05), greatest mean mitotic rate (*P* < 0.05), and greatest proportion of Ki67% > 14. Five patients developed distant recurrence (7%): three from the intermediate-risk group (7%), one from the low-risk group (10%), and one from the high-risk group (6%).

**Conclusion:**

This is the first Australian study reporting the experience with medium-term recurrence outcomes of using the Oncotype DX assay in breast cancer. Chemotherapy was rarely given for patients with low-to-intermediate RS and always offered in high RS. This pattern of prescribing was associated with low rates of distant recurrence. National funding models should be considered.

## 1. Introduction

Patients with the estrogen receptor-positive (ER+), human epidermal growth factor receptor 2-negative (HER2−) phenotype represent the most common variety of breast carcinoma, accounting for 75% of all breast cancers [1, 2]. The widespread use of adjuvant chemotherapy in this subtype has contributed to the reduction of breast cancer-related mortality. However, not all patients benefit from adjuvant chemotherapy. Adjuvant chemotherapy is associated with significant morbidity and expense, and its avoidance for patients identified at lower risk where there is minimal benefit is ideal [3]. Traditional histological parameters are of variable reliability as predictors of risk in a significant proportion of ER+/HER2− patients. Genomic assays and algorithms have been developed to quantify expression of specific genes that play an important role in the recurrence risk of breast cancer and more accurately determine the likelihood of benefit of adjuvant chemotherapy [3].

The Oncotype DX test is a genomic assay that generates a Recurrence Score (RS) predicting the 10-year risk of recurrence and response to adjuvant chemotherapy in ER+/HER2− early breast cancer patients. It comprises sixteen cancer-related genes, selected based on their statistical association with tumor proliferation, invasion, and distant breast recurrence, as well as five reference “house-keeping” control genes [4]. An algorithm generates an RS between 0−100, where higher scores indicate a higher probability of distant recurrence with adjuvant tamoxifen therapy alone [5]. Initially, patients were grouped into three risk categories: low risk (RS < 18), intermediate risk (RS 18–30), and high risk (RS > 30). However, the benefit of adjuvant chemotherapy remained unclear in the intermediate-risk group of patients. The prospective Trial Assigning Individualized Options for Treatment (TAILORx) involved 10,273 women with ER+/HER2−, node-negative (N0) breast cancer [6]. Of the intermediate-risk RS group, 3458 patients were randomized to receive adjuvant endocrine therapy alone, and 3449 patients were assigned adjuvant chemoendocrine therapy [6]. After a 9-year follow-up, this trial demonstrated low distant recurrence risk for RS 0–10 and no benefit from adjuvant chemotherapy among women over 50 years of age with intermediate RS 11–25 [6, 7]. At 9 years in the intermediate-risk RS group, the rate of freedom from distant recurrence was 94.5% and 95% in the endocrine and chemoendocrine therapy groups, respectively. There was no significant difference in disease-free survival or local recurrence rates [6]. For those individuals with high RS 26–100 and women under 50 years of age with intermediate RS 16–25, adjuvant chemotherapy significantly reduced cancer recurrence and mortality [6].

Our study examined the adjuvant systemic therapy prescribing patterns and distant recurrence rates for ER+/HER2− nonmetastatic breast cancer patients in a multidisciplinary Australian institutional setting based on the Oncotype DX recurrence risk groups.

## 2. Materials and Methods

A single-institution, retrospective case series of all patients from a prospectively designed database who underwent Oncotype DX assay testing between February 2012 and June 2016 was conducted at Sydney Adventist Hospital following definitive surgery. The primary aim was to determine breast cancer distant recurrence. These were correlated with adjuvant chemoendocrine prescribing patterns and the Oncotype DX RS (Genomic Health, Inc). Clinicopathological data were acquired from hospital and clinic records. The inclusion criteria were all ER+/HER2− nonmetastatic breast cancer patients who had Oncotype DX recurrence score performed as recommended by the multidisciplinary team (MDT). Both node-positive and node-negative patients were included.

Histopathological data collected included tumor type, maximum histological size, Ki67 (%), ER/PR/HER2 receptor characteristics (ER/PR positivity was defined as >1% cells staining by immunohistochemistry), and nodal status. The size of multifocal tumors was measured using the largest histological focus. Ki67 analysis divided patients into Ki67 ≤ 14% and Ki67 > 14% [7, 8]. Oncotype DX RS was collected, and patients were divided into low-risk (RS < 11), intermediate-risk (RS 11–25), and high-risk (RS > 25) groups, consistent with the TAILORx study categorization. The type and duration of adjuvant treatment was determined at a regular breast multidisciplinary team (MDT). Follow-up data were obtained up to April 2021, with follow-up calculated from the date of initial breast cancer surgery to the date of last follow-up. One patient lost to follow-up at 12 months without evidence of disease after moving overseas was censored from follow-up analysis.

The statistical analysis was prespecified. Continuous variable data were reported as either median or mean with standard deviation. Student's *t*-test was used to compare patient groups. A chi-square test was used to compare categorical data. For all analyses, a *P* value ≤ 0.05 was considered statistically significant. Statistics were calculated using Microsoft Excel 2010 (Redmond, WA, USA).

## 3. Results

### 3.1. Patient Characteristics

All 71 included patients were female. The majority of the cohort patients were classified into the intermediate-risk RS group (n = 45, 63%), followed by the high-risk RS group (n = 16, 23%) and then the low-risk RS group (n = 10, 14%). The median follow-up for live patients was 6.1 years (range 4–8.9 years). 70 patients (99%) had five or more years of follow-up, and one patient (1%) had four years of follow-up.

Table 1 demonstrates patient and tumor characteristics for the cohort. The mean age at diagnosis was 56 years (range, 33–77). Seventy-two percent of the patients had a wide local excision (WLE) with sentinel lymph node biopsy (WLE + SLNBx), with IDC accounting for the majority of the histopathology (87%). Most of the tumors were between 10–20 mm in size (52%) and grade 2 (68%). All patients were ER positive. Lymphovascular invasion (LVI) was present in 28% of cases. 71% of patients had a Ki67 > 14%. In regards to the axillary nodal status, 52% were pN0, 45% pN1, 1.5% pN2, and 1.5% had pN3 disease. Extranodal spread was present in 37% of patients with node-positive disease.

Varying ranges of clinicopathological characteristics were observed between the three RS subgroups (Table 1). Mean age was similar between the groups. The high-risk RS group had the smallest mean tumor size, greatest mean grade (*P* < 0.05), lowest mean ER%, lowest mean PR%, greatest mitotic rate (*P* < 0.05), and greatest proportion of Ki67% > 14. The low-risk RS group had the largest mean tumor size, the greatest representation of invasive lobular carcinoma (ILC), and the highest mean ER/PR% (*P* < 0.05).

### 3.2. Adjuvant Treatment and Follow-Up Data

The high-risk RS group received the highest proportion of endocrine and chemotherapy (94%). The majority of the intermediate-risk RS group received endocrine treatment only (96%), with only one patient receiving endocrine and chemotherapy (2%) and another patient declining any adjuvant systemic therapy (2%) (Table 2).

Five patients developed distant metastasis from the cohort (7%) with one from the low-risk RS group, three from the intermediate-risk RS group, and one from the high-risk RS group (Table 3). Two patients (3%) died from metastatic disease. The absolute risk of metastasis was 10% in the low-risk RS group, 7% from the intermediate-risk RS group, and 6% from the high-risk RS group.

In the high-risk RS group, all but one patient received chemotherapy. The one patient who did not receive chemotherapy was a 53-year-old patient who had declined adjuvant chemotherapy and remains disease free after 5 years of follow-up. She initially had a WLE + SLNBx for a 35 mm IDC, grade 3, Ki 67 30%, ER 80%, PR 0%, pN0, Oncotype RS 40, and completed adjuvant endocrine therapy and radiotherapy.

## 4. Discussion

Since the development of Oncotype DX RS assay in 2004, RS testing has allowed for a more nuanced understanding of tumor biology and potential benefit from adjuvant chemotherapy in certain patient groups [9]. It has also led to the reduction of the use of adjuvant chemotherapy as demonstrated in a study by Hassett et al., reporting a 13% decline in the use of chemotherapy between 2006 and 2008 [10]. Since that time, RS testing has been incorporated into international practice guidelines and now forms the backbone of the prognostic staging groups presented in the 8th edition of the AJCC Cancer Staging manual [11]. A study by de Boer et al. showed that the Oncotype DX changed multidisciplinary treatment recommendations by 24% [12]. In a recent Australian study including our institution, Oncotype DX changed treatment recommendations in 38% of patients, deescalating 65% from chemotherapy to hormonal therapy and adding chemotherapy to 14% who would otherwise have had adjuvant endocrine therapy [13]. In our series, most patients had a low or intermediate RS score (n = 55) and 54 of the 71 patients (76%) avoided chemotherapy (Table 2).

The prescribing patterns illustrated in this study show that the low- and intermediate-risk RS patients received adjuvant endocrine therapy only and high-risk RS patients received adjuvant chemoendocrine therapy. Patients in the high-risk RS group would appear to have gained benefit from adjuvant chemotherapy given there was only one recurrence in this group despite their higher risk of disease after at least 5 years of follow-up. Patients in the low-risk RS groups on the other hand avoided chemotherapy with a systemic recurrence rate of 6%. This recurrence rate is consistent with the expected risk of relapse and confirms that this group was one for which the addition of chemotherapy would unlikely further reduce the rate of recurrence.

Prior to the TAILORx study, the approach to adjuvant therapy for the intermediate-risk RS group remained uncertain, given the higher rate of breast cancer recurrence with potentially a small benefit from adjuvant chemotherapy [14]. TAILORx demonstrated that, for the intermediate-risk RS group, endocrine therapy was noninferior to chemoendocrine therapy in invasive disease-free survival or death after nine years of follow-up [6]. The findings of our study are consistent with those of TAILORx. In the TAILORx study, there was approximately 95% freedom from recurrence at distant sites at 9 years, while in our study, it was similar at 93% after a median follow-up of 6.1 years, noting that our study also included node-positive patients, accounting for half of the recurrences.

The chemotherapy prescribing patterns were similar to those in a study by Stemmer et al. based on an Israeli cancer registry where Oncotype DX is routinely funded, involving 1365 ER+/HER2−, node-negative breast cancer patients [15]. Chemotherapy use was consistent with the RS, with 0%, 9.4%, and 69.9% receiving adjuvant chemotherapy for patients with RS 0–10, 11–25, and ≥ 26, respectively [15]. Patients with low- and intermediate-risk RS and receiving adjuvant endocrine treatment only had a 10-year distant breast cancer recurrence risk of 2.7%–5.7%, with no statistical difference between chemotherapy treated and untreated patients [15]. This supported the use of endocrine therapy alone in this group of patients [15].

The present study demonstrates the following clinicopathologic characteristics to be statistically significantly related to high-risk RS: higher-grade cancers, low ER/PR expression, and higher mitotic rate. Prediction of a RS > 25 may be of particular clinical interest in settings where the Oncotype Dx assay is not available, given this subgroup of patients would benefit from adjuvant chemotherapy. In a similar study by Thibodeau and Voutsadakis (2019), three pathological factors were significantly associated with an RS > 25: high grade, low positivity for ER (<90%), and low positivity for PR (<20%) [14].

Multigene panels help to personalize systemic adjuvant therapy choices. Other than Oncotype DX assay, there is a 70-gene panel (MammaPrint), 50-gene panel (Prosigna), 12-gene panel (EndoPredict), and 7-gene panel (Breast Cancer Index). To date, Oncotype DX assay testing remains the only multigene assay validated to predict the likelihood of chemotherapy benefit for node-negative patients. The Oncotype DX is preferred by the National Comprehensive cancer Network (NCCN) breast cancer panel for prognosis and prediction of chemotherapy benefit [16]. Other gene expression assays can provide prognostic information, but the ability to predict chemotherapy benefit is less substantiated.

There is less literature in regards to Oncotype DX use for node-positive patients. In this study, 48% of patients were lymph node positive. The recurrence rate in node-positive patients was not higher than in the node-negative patients, and many did not receive chemotherapy. Of the node-positive patients, 81% received adjuvant endocrine only, and 19% received chemoendocrine therapy. Genomic testing may have had an even greater impact in the node-positive cohort in identifying those patients for whom adjuvant chemotherapy would usually have been recommended but from which they would receive no benefit. This observation is supported by a study by Stemmer et al. who concluded that, in ER+/HER2− patients with micrometastasis/1–3 node-positive breast cancer with an RS ≤ 25, the 5-year recurrence rate of adjuvant chemotherapy-treated (2.3%) compared to chemotherapy-untreated (4.4%) patients was not statistically significant (*P*=0.521) [17]. RxPONDER (SWOGS1007) is a phase III trial currently analyzing whether there are benefits with adjuvant chemotherapy in N1 patients with RS ≤ 25 [18]. It intends to recruit 10,000 patients, with ER/PR-positive, HER2-negative pN1 breast cancer. Patients are randomized to receive chemoendocrine therapy versus endocrine therapy alone [18]. The first interim results presented at The San Antonio Breast Cancer Symposium 2020 demonstrated that, after a mean follow-up of 5.1 years, there was no adjuvant chemotherapy benefit in postmenopausal patients with an RS ≤ 25, however, there was a strong invasive disease-free survival for adjuvant chemoendocrine treatment in premenopausal patients [19].

Our study is limited by relatively small numbers but does represent the largest and the only published experience in Australia of routine use with outcomes of Oncotype DX genomic testing. It follows on from previous work [13] looking at the impact of genomic testing with Oncotype DX on decision making in ER+/HER2− patients with the aim of determining if our patient outcomes correlate with the outcomes predicted by the assay and whether outcomes are acceptable. The recurrence rates reported do demonstrate outcomes that match expectation. The follow-up duration in this study (median 6.1 years) is shorter than in some clinical trials but is substantial and adequate to demonstrate the reliability and safety of using Oncotype DX as a decision aid. We will continue to follow-up this patient cohort and subsequently publish longer-term follow-up outcomes (at 10 years), particularly as the risk of recurrence may persist after hormonal treatment is stopped for ER-positive tumors [20, 21].

Our study has validated that, in an Australian setting, Oncotype DX is reliable in determining prognosis and aiding chemotherapy decision making. Despite the increasing evidence and several international breast cancer guidelines recommending the use of genomic assay tests, the lack of Medicare and private health fund reimbursement means that they are seldomly utilized in Australia as the cost is prohibitive (approximately $5000 for Oncotype DX) for most patients. Without the use of genomic assay tests, many Australian patients with breast cancer receive chemotherapy with minimal benefit or do not receive chemotherapy and are put at increased risk of recurrence. Chemotherapy has a significant impact on patients through side effects and loss of income. There have been several applications over the years to the Australian Government Medical Services Advisory Committee (MSAC) for funding of genomic assay tests. In a 2019 resubmission, after considering the strength of the available evidence in relation to safety, clinical effectiveness, and cost effectiveness, the MSAC did not support Medicare funding for Oncotype DX testing, citing that there was insufficient evidence to identify patients who would benefit from chemotherapy or be spared chemotherapy [22]. In other countries such as the United States, all major insurers cover the cost of genomic assay tests for eligible patients with early breast cancer. If we are to provide the best medical care, national funding needs to be available.

## 5. Conclusions

This is the first Australian published study reporting the utility and medium-term recurrence outcomes of the Oncotype DX assay for early breast cancer. Chemotherapy was rarely given to patients with low- and intermediate-risk RS with low rates of systemic recurrence and offered to all women in high-risk RS groups with excellent systemic disease control.

## Figures and Tables

**Table 1:** Patient and tumor characteristics based on recurrence score.

	All patients, *n* = 71	RS < 11, *n* = 10	RS 11–25, *n* = 45	RS > 25, *n* = 16	*P* value (low/intermediate risk vs. High risk)
*Age*					
Mean (SD), years	56 (9)	59 (11)	54 (10)	57 (6.9)	0.52

*Age category, n (%)*					
≤50 years	23 (32)	3 (30)	17 (38)	3 (19)	—
51–65 years	34 (48)	4 (40)	19 (42)	11 (69)	—
66–79 years	14 (20)	3 (30)	9 (20)	2 (12)	—

*Type of operation, n (%)*					
WLE + SLNBx	52 (72)	8 (80)	32 (71)	12 (75)	—
WLE + ALNDx	9 (13)	1 (10)	5 (11)	3 (19)	—
Mx + SLNBx	7 (10)	0	6 (13)	1 (6)	—
Mx + ALNDx	3 (5)	1 (10)	2 (5)	0	—

*Focality of tumor, n (%)*					
Unifocal	60 (85)	8 (80)	37 (82)	15 (94)	0.24
Multifocal	11 (15)	2 (20)	8 (18)	1 (6)	

*Histology, n (%)*					
IDC	62 (87)	7 (70)	39 (87)	16 (100)	—
ILC	8 (11)	3 (30)	5 (11)	0	—
Papillary	1 (2)	0	1 (2)	0	—

*Tumor size in the greatest dimension*					
Mean (SD), mm	19.49 (13.81)	26.4 (31.13)	19.96 (9.84)	17.19 (9)	0.11

*Tumor size category, n (%)*					
≤10 mm	11 (15)	2 (20)	3 (7)	6 (38)	—
>10–20 mm	37 (52)	4 (40)	28 (62)	5 (31)	—
>20–30 mm	15 (21)	2 (20)	10 (22)	3 (19)	—
>30 mm	8 (12)	2 (20)	4 (9)	2 (12)	—

*Tumor grade category, n (%)*					
Grade 1	5 (7)	1 (10)	4 (9)	0 (0)	—
Grade 2	48 (68)	8 (80)	35 (78)	5 (31)	—
Grade 3	18 (25)	1 (10)	6 (13)	11 (69)	—

*Average grade*					
Mean (SD)	2.17 (0.54)	2 (0.47)	2.04 (0.46)	2.69 (0.48)	<0.001

*Nodal stage, n (%)*					
N0	37 (52)	3 (30)	25 (56)	9 (56)	—
N1 (1–3)	32 (45)	6 (60)	19 (42)	7 (44)	—
N2 (4–9)	1 (1.5)	0 (0)	1 (2)	0 (0)	—
N3 (>10)	1 (1.5)	1 (10)	0 (0)	0 (0)	—

*Size of the largest nodal disease (mm)*					
Mean (SD)	5.56 (5.36)	2.32 (2.71)	4.35 (5.54)	4.28 (5.54)	0.88

*Extranodal spread, n (% of patients with positive nodes)*					
Present	11 (37)	3 (50)	7 (41)	1 (17)	0.25

*ER %*					
Mean (SD)	85 (11)	89 (8.4)	85 (11)	79 (12)	0.01

*PR %*					
Mean (SD)	62.2 (34.3)	91 (5.16)	62.3 (32.8)	35.1 (37.6)	<0.001

*Mitotic rate*					
Mean (SD)	9.25 (8.3)	6.5 (5.76)	6.88 (3.87)	15.81 (12.03)	<0.001

*Lymphovascular invasion, n (%)*					
Present	20 (28)	6 (60)	9 (20)	5 (31)	0.25

*Ki67%, n (%)*					
≤14%	20 (29)	3 (33)	15 (34)	2 (12)	0.1
>14%	49 (71)	6 (66)	29 (66)	14 (88)	

*Adjuvant radiotherapy, n (%)*					
Received	57 (80)	10 (100)	34 (76)	13 (81)	0.91

WLE, wide local excision; Mx, mastectomy; SLNBx, sentinel lymph node biopsy; ALNDx, axillary lymph node dissection; IDC, invasive ductal carcinoma, ILC, invasive lobular carcinoma.

**Table 2:** Adjuvant chemoendocrine therapy based on recurrence score.

	All patients *n* = 71	RS < 11 *n* = 10	RS 11–25 *n* = 45	RS > 25 *n* = 16
Type of adjuvant therapy, *n* (%)				
No endocrine therapy or chemotherapy	1 (2)	0 (0)	1 (2)	0 (0)
Endocrine only	54 (76)	10 (100)	43 (96)	1 (6)
Endocrine and chemotherapy	16 (22)	0 (0)	1 (2)	15 (94)

**Table 3:** Characteristics of patients developing metastatic disease.

Patient (age at the time of surgery)	Oncotype DX RS	Operation	Pathology	Adjuvant treatment	Recurrence
Patient 1 (40 years)	7 low risk	WLE and SLNBx	13 mm IDC, grade 2, Ki 67 10%, ER 100%, PR 100%, pN0	Endocrine (ceased after 12 months due to side effects) and radiotherapy	Metastatic disease 7 years after surgery
Patient 2 (52 years)	13 intermediate risk	WLE and SLNBx	15 mm IDC, grade 2, Ki 67 40%, ER 90%, PR 70%, pN0	Endocrine and radiotherapy	Metastatic disease 5 years after surgery
Patient 3 (50 years)	23 intermediate risk	Mastectomy and ALNDx	18 mm IDC, grade 3, Ki67 20%, ER 90%, PR 80%, pN1 8 mm sentinel node deposit with no extranodal spread	Endocrine	Metastatic disease 3 years after surgery and died in year 4
Patient 4 (60 years)	15 intermediate risk	Mastectomy and SLNB	50 mm ILC, grade 2, Ki 67 15%, ER 80%, PR 70%, pN1 3 mm sentinel node deposit with extranodal spread	Endocrine and radiotherapy	Metastatic disease 2 years after surgery and died in year 4
Patient 5 (65 years)	35 high risk	WLE and ALNDx	35 mm IDC, grade 3, Ki 67 49%, ER 90%, PR 0%, pN1	Chemotherapy, endocrine, and radiotherapy	Metastatic disease 6 years after surgery

WLE, wide local excision; SLNBx, sentinel lymph node biopsy; ALNDx, axillary lymph node dissection; IDC, invasive ductal carcinoma, ILC, invasive lobular carcinoma; ER, estrogen receptor positive; PR, progesterone receptor positive; pN, pathological nodal status.

## Data Availability

Data supporting the results of the study can be made available on request.

## Abbreviations

ER: Estrogen receptor positive
PR: Progesterone receptor positive
HER2: Human epidermal growth factor receptor 2
RS: Recurrence score
TAILORx: Trial Assigning Individualized Pptions for Treatment
WLE: Wide local excision
Mx: Mastectomy
SLNBx: Sentinel lymph node biopsy
ALNDx: Axillary lymph node dissection
IDC: Invasive ductal carcinoma
ILC: Invasive lobular carcinoma
LVI: Lymphovascular invasion
pN: Pathological nodal status
MSAC: Australian Government Medical Services Advisory Committee.
